# Genome-wide association study revealed genomic regions related to white/red earlobe color trait in the Rhode Island Red chickens

**DOI:** 10.1186/s12863-016-0422-1

**Published:** 2016-08-05

**Authors:** Changsheng Nie, Zebin Zhang, Jiangxia Zheng, Hongyan Sun, Zhonghua Ning, Guiyun Xu, Ning Yang, Lujiang Qu

**Affiliations:** 1 Department of Animal Genetics and Breeding, College of Animal Science and Technology, China Agricultural University, Beijing, 100193 China; 2 College of Animal Science and Technology, Yangzhou University, Yangzhou, Jiangsu 225009 China

**Keywords:** Rhode Island Red chicken, Earlobe color, GWAS

## Abstract

**Background:**

Earlobe color is a naturally and artificially selected trait in chicken. As a head furnishing trait, it has been selected as a breed characteristic. Research has demonstrated that white/red earlobe color was related to at least three loci and sex-linked. However, there has been little work to date to identify the specific genomic regions and genes response to earlobe color in Rhode Island Red chickens. Currently, it is possible to identify the genomic regions responsible for white/red earlobe in Rhode Island Red chicken to eliminate this gap in knowledge by using genome-wide association (GWA) analysis.

**Results:**

In the present study, genome-wide association (GWA) analysis was conducted to explore the candidate genomic regions response to chicken earlobe color phenotype. Hens with red dominant and white dominant earlobe was used for case-control analysis by Illumina 600 K SNP arrays. The GWA results showed that 2.38 Mb genomic region (50.13 to 52.51 Mb) with 282 SNPs on chromosome Z were significantly correlated to earlobe color, including sixteen known genes and seven anonymous genes. The sixteen genes were *PAM*, *SLCO4C1*, *ST8SIA4*, *FAM174A*, *CHD1*, *RGMB*, *RIOK2*, *LIX1*, *LNPEP*, *SHB*, *RNF38*, *TRIM14*, *NANS*, *CLTA*, *GNE*, and *CPLX1*.

**Conclusions:**

The study has revealed the white/red earlobe trait is polygenic and sex-linked in Rhode Island Red chickens. In the genome significant ~2.38 Mb region, twenty-three genes were found and some of them could play critical roles in the formation of white/red earlobe color, especially gene *SLCO4C1*. Taken together, the candidate genes findings herein can help elucidate the genomic architecture of response to white/red earlobe and provide a new insight on mechanisms underlying earlobe color in Rhode Island Red chickens and other breeds.

**Electronic supplementary material:**

The online version of this article (doi:10.1186/s12863-016-0422-1) contains supplementary material, which is available to authorized users.

## Background

Earlobe color, a qualitative trait in chicken, is artificially and naturally selected in various breeds [1, 2]. It is a part of skin structure on the face without feathers and below the ear. In nature, shining color is selected to get more attention of their predators or partners [3, 4]. Earlobe color, a head furnishing trait, has been selected as a breed characteristic. Red or white earlobes are predominant in a number of the wild and domestic chickens worldwide [2] although yellow, blue, purple and black earlobe could be found in some breeds.

Variation in chicken earlobe color may be caused by ancestral lineages and mutations [2], as well as the adaptability to local conditions [5]. For example, The presence of white earlobes is due to purine base deposition and the formation of other color earlobe is attributed to the mixture of different pigments including melanin or carotenoid [6]. The red earlobe, the same color as the rest of the red face, could reflect the health of the birds with the degree of redness [7].

Genetic foundation studies of chicken earlobe color were conducted in previous studies. The white earlobe has been identified to be polygenic and it appeared to be sex-linked in some breeds [7]. The mottled earlobe in Rhode Island Reds appears to be produced by two recessive autosomal genes [8]. Recently, Wragg et al. [9] identified 7 concordant significances (*P* < 0.05 and Z > 4) SNPs on chicken chromosomes 1, 2, 4 and Z related to white/red earlobe color by using genome-wide association (GWA) analysis, indicating that earlobe color trait is sex-linked and polygenic. However, there has been little work to date to identify the accurate genomic regions and genes response to earlobe color in Rhode Island Red chickens. To eliminate this gap in knowledge, we performed a genome-wide association study (GWAS) to identify the genomic regions responsible for white/red earlobe in the Rhode Island Red chickens.

## Results

After quality control, a total of 78 female chickens aged 20 weeks old were analyzed, of which 48 (61 %) presented white earlobe color as cases and 30 (39 %) presented red earlobe color as controls. The MultiDimensional scaling (MDS) analysis indicated the absence of population stratification in our study population (Additional file 1: Figure S1).

**Fig. 1:**
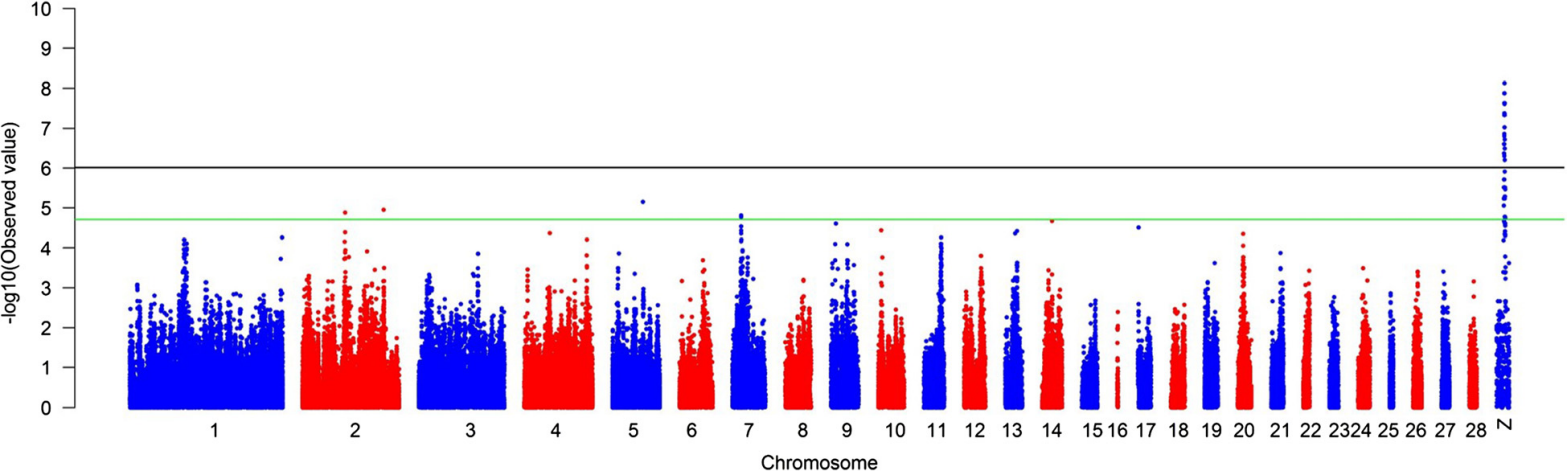
Manhattan plot of genome wide association study for white/red earlobe color. The Manhattan plot indicates -log10 (observed P-values) for genome-wide SNPs (y-axis) plotted against their respective positions on each chromosome (x-axis), and the horizontal green and black lines indicate the suggestive significant (1.95 × 10–5) and genome-wide significant (9.81 × 10–7) threshold, respectively

Based on the Manhattan plot for earlobe color, we observed a total of 282 significantly associated SNPs spanning from 50.13 to 52.51 Mb (~2.38 Mb) on chromosome Z (P value < 9.81 × 10^−7^) in the sexually mature hens (Fig. 1 and Additional file 2: Table S1). The linkage disequilibrium plot (Additional file 3: Figure S2) showed the detected SNP markers were strongly linked in a haplotype block. Moreover, in this ~2.38 spanning, twenty-three genes were found related to earlobe color phenotype, including sixteen annotated genes and seven anonymous genes: *PAM*, *SLCO4C1*, *ST8SIA4*, *FAM174A*, *CHD1*, *RGMB*, *RIOK2*, *LIX1*, *LNPEP*, *SHB*, *RNF38*, *TRIM14*, *NANS*, *CLTA*, *GNE*, *CPLX1*, *LOC107052343*, *LOC107052344*, *LOC107052345*, *LOC101752070*, *LOC107052346*, *LOC100857660* and *LOC101752249*. The sixteen annotated genes and their functions were displayed in Table 1.

**Table 1:** Summary description of genes in ~2.38 Mb region on chromosome Z

Gene	Full Name	Location (bp)^a^	Length (bp)^a^	Number (SNP)^b^	Functions
*PAM*	Peptidylglycine alpha-amidating monooxygenase	50,043,929 - 50,172,820	128,892	7	Catalyze neuroendocrine peptides to active alpha-amidated products [17, 30], type 2 diabetes [13].
*SLCO4C1*	Solute carrier organic anion transporter family member 4C1	50,256,470–50,283,287	26,818	2	Transport estrone 3-sulfate [31].
*ST8SIA4*	ST8 alpha-N-acetyl-neuraminide alpha-2,8-sialyltransferase 4	50,646,216–50,699,691	53,476	0	Modulator of the adhesive properties of neural cell adhesion molecule [16].
*LOC107052343*	NA	50,802,139–50,808,822	6,684	0	NA
*FAM174A*	Family with sequence similarity 174 member A	50,808,437–50,822,236	13,800	0	NA
*CHD1*	Chromodomain helicase DNA binding protein 1	51,274,317–51,322,737	48,421	0	Disease related [12, 32, 33, 34].
*RGMB*	Repulsive guidance molecule family member b	51,334,306–51,349,990	15,685	0	Angiogenesis [35], breast cancer [14].
*RIOK2*	RIO kinase 2	51,538,065–51,550,090	12,026	0	Mitotic progression [36], cytoplasmic maturation [18].
*LIX1*	Limb and CNS expressed 1	51,552,825–51,581,108	28,284	0	Fat signaling, marker for cerebral structures [19, 37].
*LNPEP*	Leucyl/cystinyl aminopeptidase	51,598,066–51,651,403	53,338	13	Vasopressin clearance and serum sodium regulation [38], associated with psoriasis [39].
*LOC107052344*	NA	51,667,644–51,675,321	7,678	1	NA
*SHB*	Src homology 2 domain containing adaptor protein B	51,704,167–51,777,551	73,385	14	Regulates cell motility [40, 41].
*LOC107052345*	NA	51,759,503–51,766,981	7,479	4	NA
*RNF38*	Ring finger protein 38	51,958,739–51,993,024	34,286	9	Disease related [15, 42].
*LOC101752070*	NA	51,999,295–52,003,026	3,732	0	NA
*TRIM14*	Tripartite motif containing 14	52,003,922–52,011,357	7,436	3	A mediator in the immune response against viral infection [43].
*NANS*	N-acetylneuraminic acid synthase	52,013,130-52,022,024	8,895	3	Change expression in response to androgen [44].
*CLTA*	Clathrin light chain A	52,040,355–52,055,158	14,804	2	Main structural component of the lattice-type cytoplasmic face of coated pits and vesicles [22, 23].
*GNE*	Glucosamine (UDP-N-acetyl)-2-epimerase	52,058,958–52,096,177	37,220	10	GNE myopathy [45], regulator of sialic acid synthesis [46].
*LOC107052346*	NA	52,149,755–52,175,567	25,813	9	NA
*LOC100857660*	NA	52,178,755–52,205,231	26,477	6	NA
*CPLX1*	Complexin 1	52,264,659–52,368,798	104,140	18	Synaptic vesicle exocytosis, bind syntaxin, part of the SNAP receptor [21, 47, 48].
*LOC101752249*	NA	52,471,803–52,483,455	11,653	2	NA

*NA* not available

^a^ Source: Reference Gallus_gallus-5.0 primary assembly (NCBI)

^b^ The number of genome significant SNPs located in gene

Additionally, a total of four autosomal regions with 6 SNPs were suggestive significantly related to white/red earlobe in chicken (1.95 × 10^−5^) (Fig. 1 and Additional file 4: Table S2), of which two on chromosome 2 (one with 2 SNPs and the other one with 1 isolated SNP) (67.19 Mb ~ 67.21 Mb), one on chromosome 5 (1 isolated SNP), and one on chromosome 7 (2 SNPs) (10.09 Mb ~ 10.12 Mb).

## Discussion

The aims of this study were to identify and estimate the genomic regions responsible for white/red earlobe in Rhode Island Red chickens, and to locate positional candidate genes association with color earlobe by using a 600 K SNP panel for genotyping. In a brief, a total of 282 genome significantly SNP markers on chromosome Z were detected in this study, which corresponded to sixteen known genes and seven anonymous genes.

Gene *SLCO4C1* (solute carrier organic anion transporter family member 4C1) was identified for earlobe color within an average of 101 kb of twenty-five the genome-wide significant SNPs (Additional file 2: Table S1). This plausible positional candidate gene, *SLCO4C1*, may have its special function in earlobe color formation as it is among numerous significant SNPs that in linkage disequilibrium (LD) building up to a QTL peak.

Candidate gene, *SLCO4C1*, belongs to the organic anion transporting polypeptide (OATP) family. Researchers have demonstrated that OATP family had the function of transportation of the amphipathic organic compounds, like bile salt in mammals [10, 11]. The component of bile salt, biliverdin, can be deposited to form blue eggshell [12]. Wang et al. [12] reported in chicken *SLCO1B3* was in response to blue eggshell color formation via transferring biliverdin. Although the molecule transfer mechanism of earlobe color formation remains unknown, earlobes color was occurred due to purine base deposition or mixture of different pigments [6]. In practical, breeders believed some relations exist between earlobe and eggshell color, which might be caused by the close association of the determining genes on the chromosome or single factor that controls the pigmentation of both egg and earlobe [7].

Thus, based on the current results, pigments deposition to form earlobe color, as well as the relations between earlobe and eggshell color, it is reasonable for us to speculate *SLCO4C1* may play an important role in the formation of earlobe color. Future validation of this assumption of *SLCO4C1* gene function is warranted in chicken.

Besides the crucial positional candidate gene *SLCO4C1*, other candidate genes located in this ~2.38 Mb region have various functions (Table 1). For example, *RNF38*, *CHD1*, *PAM*, *RGMB*, *LNPEP*, *RNF38*, *TRIM14* and *GNE* were detected to be associated with diseases [12–15]. *ST8SIA4*, *PAM*, *RIOK2*, *LNPEP*, *LIX1*, *SHB*, *TRIM14* and *GNE* have been discovered to be important in regulating the life process [16–20]. *NANS* can change expression in response to androgen. *CPLX1* and *RIOK2* were correlated with cytoplasmic maturation [18] or synaptic vesicle exocytosis [21]. *CLTA* was found associated with cytoplasmic face of coated pits [22] and vesicles progress [23]. Currently, the mechanism underlying earlobe color is almost unknown and few literatures support these candidate genes were directly in response to earlobe color. However, the GWAS results in this study may provide a clue for researcher to identify the relationship between these candidate genes and earlobe color. Further validation experiment of these genes was needed to perform.

Compared to our findings, Wragg et al. [9] has found 7 SNPs significantly associated with white/red earlobe color in various breeds via GWAS. In the study of Wragg et al., two SNPs (rs14170217 and rs14170463) were located on chromosome 2 at region 41.69 Mb and 41.89 Mb, respectively [9], which were ~25.5 Mb and ~25.3 Mb, respectively, downstream of our suggestive significantly SNPs (rs315420052 and rs313803643). The SNPs, rs14170217 and rs14170463, in Wragg et al. study were in the intron of genes, *ATP2C1* and *MRPL3*, respectively. In our study, both rs315420052 and rs313803643 were in the intron of gene *GMDS*. Another SNP, rs14762712, on chromosome Z at 32.08 Mb in Wragg et al. study [9] was also in intron in *BNC2* that important to pigment pattern formation. In summary, all detected significant SNPs in Wragg et al. study were in the intron area of genes. Only *BNC2* was more related to earlobe color, which is not found in our study.

Several reasons made the different results between ours and Wragg et al. study. Firstly, Wragg et al. use traditional breeds, Kenyan, Ethiopian, and Chilean village chickens to perform association analysis. Secondly, the phenotypic traits in Wragg et al. study were not only earlobe but yellow skin, oocyan, rose comb, and duplex comb. Thirdly, the experimental population birds almost have been post-prune in earlobe color in Wragg’s study. Moreover, compared to Wragg’s materials, our inbreeding population has a different phenotype in earlobe (Additional file 5: Figure S3) but a consistent genetic background, which is a good choice to perform GWAS. Also, GWAS always show different results in different populations [24]. Taken together, it is not surprise to obtain different results from ours and Wragg et al. study. However, both Wragg et al. and our study showed polygenic and sex-linked inheritance pattern determined earlobe color although different breeds were used in different studies.

## Conclusions

This study has revealed 282 genome significantly SNPs spanning ~2.38 Mb region on chromosome Z associated with earlobe color in Rhode Island Red chickens, corresponding to twenty-three genes. The genomic regions that we identified contain twenty-three genes with functions that suggest a role in response to earlobe color and, thus, these genes are both positional and functional candidates. Notably, among these twenty-three genes, *SLCO4C1* may play critical roles in the formation of white/red earlobe color. Taken together, the candidate genes findings herein can help elucidate the genomic architecture of response to white/red earlobe and provide a new insight on mechanisms underlying earlobe color in Rhode Island Red chickens and other breeds.

## Methods

### Animals and phenotypic data

**Fig. 2:**
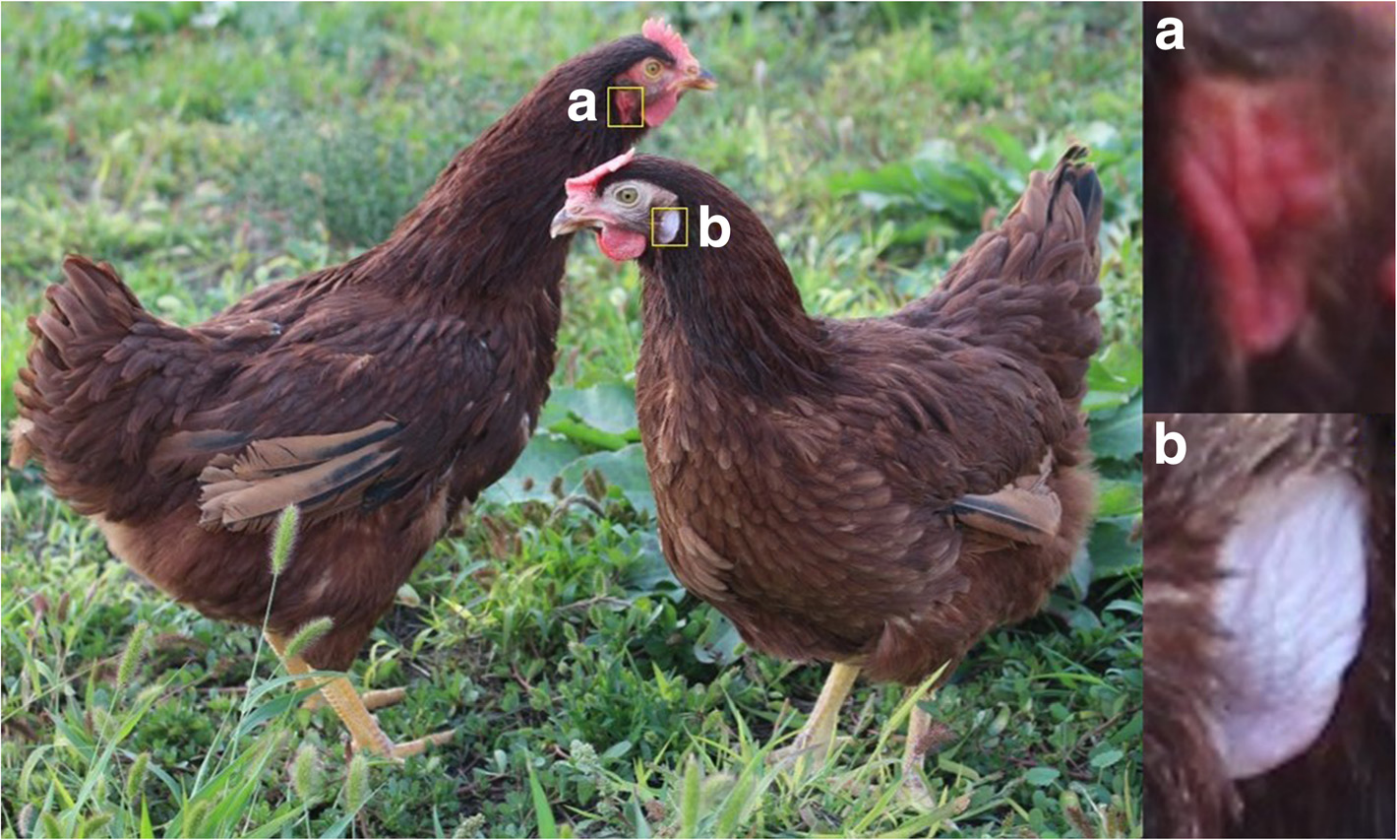
Rhode Island Red chicken hens with the white or red earlobe color. **a** Red earlobe chicken, (**b**) white earlobe chicken.

A total of 78 adult females Rhode Island Red chicken (20 weeks) were selected herein including 30 with red or predominantly red earlobe and 48 with white or predominantly white earlobe to perform GWAS (Fig. 2). In Rhode Island Red chicken, earlobe color trait is sex-linked. Males mainly had red earlobe whereas female had four grades of earlobe color: red, predominately red, white, and predominately white (Additional file 5: Figure S3). Animals have different phenotypes with a consistent genetic background is a good choice to do GWAS. Therefore, we chose sexually mature female chickens as experimental material to identify the genomic architecture of response to earlobe color in this study. In this experimental population, predominately red (Additional file 5: Figure S3b) or predominately white (Additional file 5: Figure S3c) is shown that red or white color overspread the majority of the earlobe surface. Blood samples were collected by standard venipuncture from a Rhode Island Red chicken population maintained at a commercial breeding farm in Beijing, China.

### Genotyping and quality control

Genomic DNA was extracted by standard phenol/chloroform method and genotyped with 600 K Affymetrix Axiom Chicken Genotyping Array (Affymetrix, Inc. Santa Clara, CA, USA). Affymetrix Power Tools v1.16.0 (APT) software was then used for quality control and genotype calling. Specifically, only samples with dish quality control (DQC) > 0.82 and call rate > 97 % were used for subsequent analysis. After sample quality control analysis, the mean concordance rate was 99.7 %.

The classical multidimensional scaling (MDS) analysis was used to detect population structure in PLINK v1.09 software [25]. By computing identical by state (IBS) scores for unlinked SNPs with r^2^ < 0.2 and using multidimensional scaling, a total of 78 samples were identified to involve in further analysis. Projection onto the two multidimensional scaling axes is shown in Additional file 1: Figure S1.

SNPs were removed with a minor allele frequency < 5 % in all samples (n = 135,166), or a P value of deviation from Hardy­Weinberg equilibrium (P_HWE_) < 1 × 10^−6^ in controls (n = 444). Ultimately, a total of 78 individuals and 370,106 SNPs were kept for the following association analysis.

### Statistical analysis

To test the association of each SNP with earlobe color, we used the basic case/control association analysis according to the manual of PLINK (v1.09). All qualified SNPs were subjected to the linkage disequilibrium via the *−−indep-pairwise 25 5 0.2* commend (PLINK) to generate a pruned subset of 50,946 linkage equilibrium SNPs.

The Bonferroni adjustment is a widely used method for correcting multiple hypothesis testing. Given the correlation between SNPs in linkage disequilibrium, the traditional Bonferroni adjustment appears to be overly conservative which key assumption is that all tests are independent [26]. Herein, the sum of independent blocks plus singleton markers was used to define the number of independent statistical tests [27, 28]. With this approach, 50,946 independent tests were suggested to determine the P value threshold. Hence, the genome-wide significant and suggestive P values were 9.81 × 10^−7^ (0.05/50946) and 1.95 × 10^−5^ (1/50946), respectively. The Manhattan plot of genome wide P values of association analysis was created by self-developed R programming codes. To further location candidate region that affect trait, we performed linkage disequilibrium (LD) analysis with genome significantly SNPs in Haploview software (v4.2) [29].

## Abbreviations

DQC, dish quality control; GWAS, genome-wide association study; HD, high density; IBS, Identical by state; MDS, MultiDimensional scaling analysis

## Additional files

Additional file 1: Figure S1. Sample structure evaluated by the top two MDS components. (1) White earlobe chicken, (2) red earlobe chicken. (JPG 319 kb) Additional file 2: Table S1. The information for SNPs significantly associated with earlobe color in Rhode Island Red chickens. (DOCX 37 kb) Additional file 3: Figure S2. Linkage disequilibrium (r2) plot association with white/red earlobe color. (PNG 2459 kb) Additional file 4: Table S2. Suggestive SNPs associated with the earlobe color phenotype in Rhode Island Red chickens. (DOC 240 kb) Additional file 5: Figure S3. Rhode Island Red chicken hens with different earlobe color. (a) Red earlobe chicken, (b) predominately red earlobe chicken, (c) predominately white earlobe chicken, (d) white earlobe chicken. (JPG 131 kb)
