# Supplementary figures and images for: Genome-wide association study revealed genomic regions related to white/red earlobe color trait in the Rhode Island Red chickens
Source: BMC Genet. 2016 Aug 5;17:115. doi: 10.1186/s12863-016-0422-1 (PMC4974732; doi:10.1186/s12863-016-0422-1)

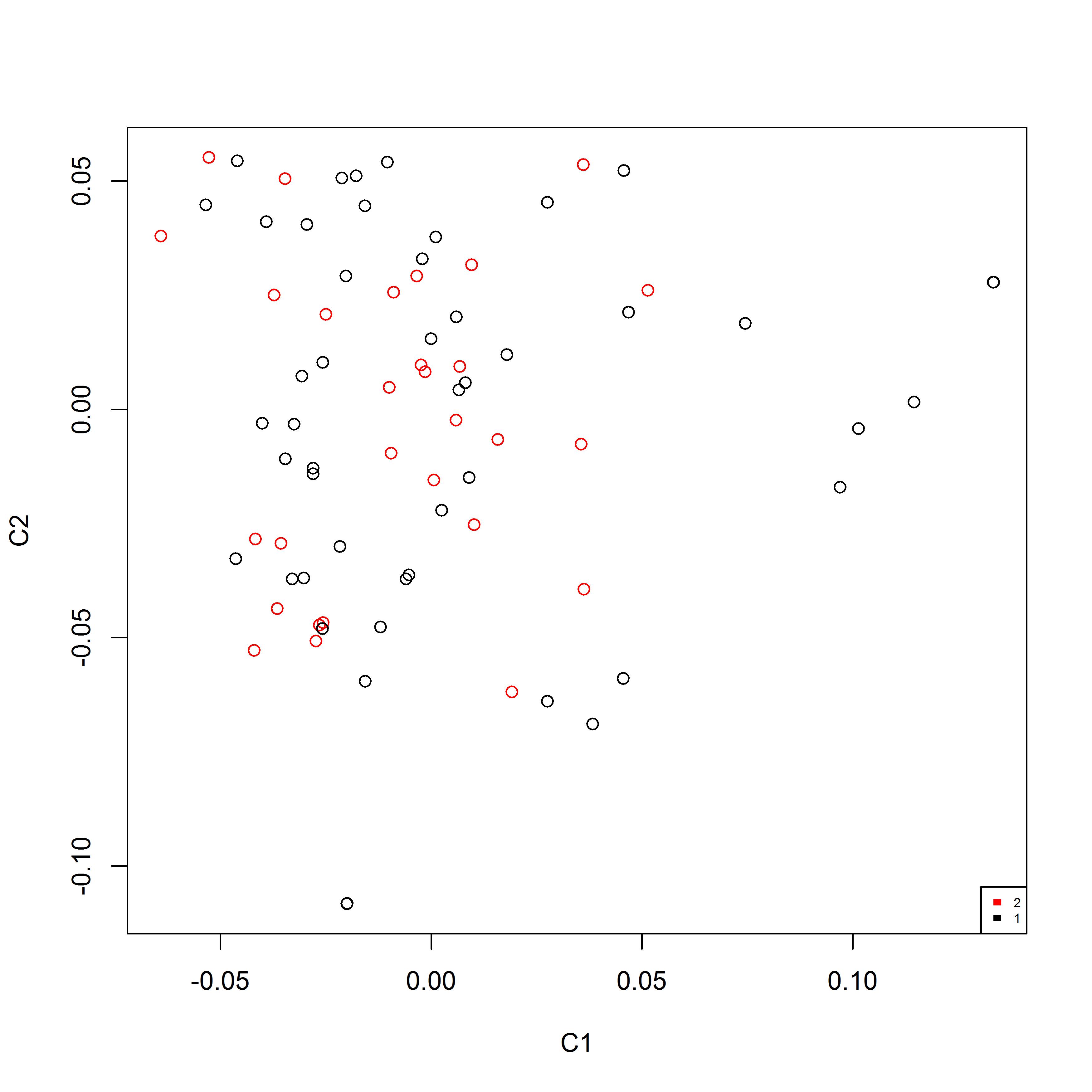

Supplement: Additional file 1: Figure S1. — Sample structure evaluated by the top two MDS components. (1) White earlobe chicken, (2) red earlobe chicken. (JPG 319 kb) [file 12863_2016_422_MOESM1_ESM.jpg]

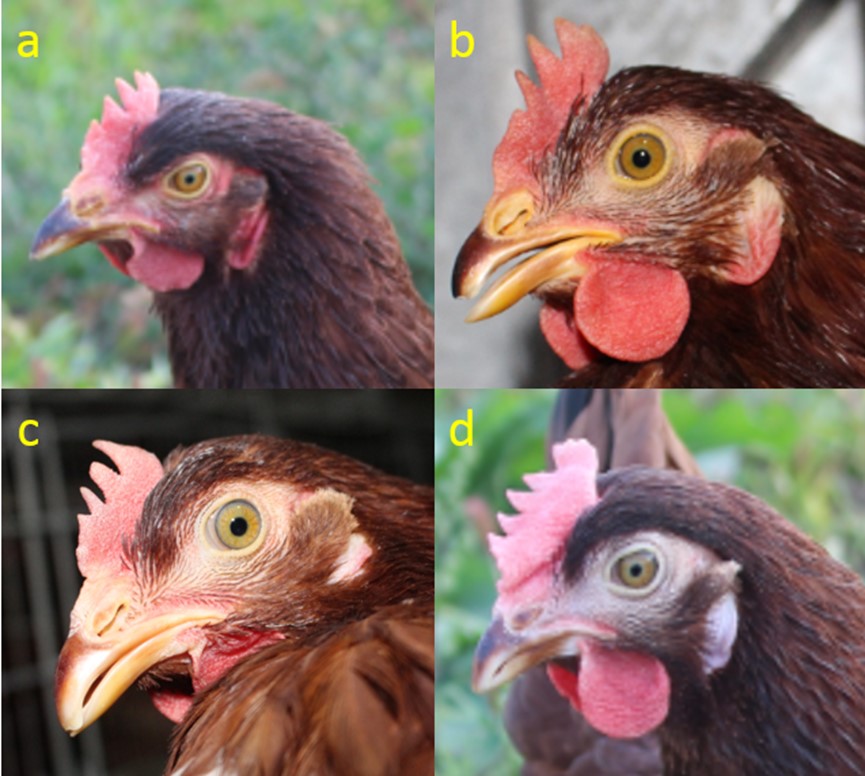

Supplement: Additional file 5: Figure S3. — Rhode Island Red chicken hens with different earlobe color. (a) Red earlobe chicken, (b) predominately red earlobe chicken, (c) predominately white earlobe chicken, (d) white earlobe chicken. (JPG 131 kb) [file 12863_2016_422_MOESM5_ESM.jpg]
