# Supplementary material for: Genome-wide association study revealed genomic regions related to white/red earlobe color trait in the Rhode Island Red chickens
Source: BMC Genet. 2016 Aug 5;17:115. doi: 10.1186/s12863-016-0422-1 (PMC4974732; doi:10.1186/s12863-016-0422-1)
Supplement: Additional file 2: Table S1. — The information for SNPs significantly associated with earlobe color in Rhode Island Red chickens. (DOCX 37 kb) [file 12863_2016_422_MOESM2_ESM.docx]

**Table S1. The information for SNPs significantly associated with earlobe color in Rhode Island Red chickens.**

| Chromosome | SNP | Position  (bp)^a^ | Minor allele | MAF | Major allele | *P-*value | Candidate/Nearest genes^a^ |
| --- | --- | --- | --- | --- | --- | --- | --- |
| Z | rs315666703 | 51846761 | A | 0.3191 | G | 7.445E-09 | *RNF38* |
| Z | rs317753838 | 51374689 | C | 0.3158 | T | 1.336E-08 | *RGMB* |
| Z | rs315529281 | 51392214 | A | 0.3158 | T | 1.336E-08 | *RGMB* |
| Z | rs317639787 | 52021208 | A | 0.3158 | T | 1.336E-08 | *NANS* |
| Z | rs313308667 | 52084074 | T | 0.3158 | C | 1.336E-08 | *GNE* |
| Z | rs312270617 | 51178063 | C | 0.3125 | G | 2.328E-08 | *CHD1* |
| Z | rs315597590 | 51219293 | A | 0.3125 | C | 2.328E-08 | *CHD1* |
| Z | rs316306388 | 51222576 | G | 0.3125 | T | 2.328E-08 | *CHD1* |
| Z | rs315080354 | 51237180 | T | 0.3125 | A | 2.328E-08 | *CHD1* |
| Z | rs312451674 | 51271979 | A | 0.3125 | C | 2.328E-08 | *CHD1* |
| Z | rs316348176 | 51363886 | T | 0.3125 | C | 2.328E-08 | *RGMB* |
| Z | rs318133017 | 51368860 | G | 0.3125 | A | 2.328E-08 | *RGMB* |
| Z | rs317179777 | 51381446 | G | 0.3125 | C | 2.328E-08 | *RGMB* |
| Z | rs16113782 | 51386239 | T | 0.3125 | C | 2.328E-08 | *RGMB* |
| Z | rs14768001 | 51391323 | C | 0.3125 | G | 2.328E-08 | *RGMB* |
| Z | rs315948164 | 51395922 | T | 0.3125 | C | 2.328E-08 | *RGMB* |
| Z | rs313045408 | 51402602 | A | 0.3125 | G | 2.328E-08 | *RGMB* |
| Z | rs16113805 | 51407269 | G | 0.3125 | A | 2.328E-08 | *RGMB* |
| Z | rs314918967 | 51418327 | T | 0.3125 | C | 2.328E-08 | *RIOK2* |
| Z | rs314006082 | 51420465 | T | 0.3125 | C | 2.328E-08 | *RIOK2* |
| Z | rs313356321 | 51431329 | T | 0.3125 | C | 2.328E-08 | *RIOK2* |
| Z | rs314971450 | 51435742 | C | 0.3125 | T | 2.328E-08 | *RIOK2* |
| Z | rs317030850 | 51449785 | T | 0.3125 | C | 2.328E-08 | *RIOK2* |
| Z | rs13768590 | 51452204 | C | 0.3125 | T | 2.328E-08 | *RIOK2* |
| Z | rs313726384 | 51458240 | G | 0.3125 | A | 2.328E-08 | *RIOK2* |
| Z | rs315898398 | 51466806 | G | 0.3125 | A | 2.328E-08 | *RIOK2, LIX1* |
| Z | rs13768598 | 51467933 | T | 0.3125 | C | 2.328E-08 | *RIOK2, LIX1* |
| Z | rs314950031 | 51471306 | T | 0.3125 | C | 2.328E-08 | *RIOK2, LIX1* |
| Z | rs16770437 | 51475419 | T | 0.3125 | C | 2.328E-08 | *RIOK2, LIX1* |
| Z | rs13813928 | 51481623 | G | 0.3125 | A | 2.328E-08 | *RIOK2, LIX1* |
| Z | rs16770452 | 51485946 | A | 0.3125 | G | 2.328E-08 | *RIOK2, LIX1* |
| Z | rs315544680 | 51486217 | G | 0.3125 | A | 2.328E-08 | *RIOK2, LIX1* |
| Z | rs314457670 | 51488167 | C | 0.3125 | T | 2.328E-08 | *RIOK2, LIX1* |
| Z | rs316175272 | 51489228 | A | 0.3125 | G | 2.328E-08 | *RIOK2, LIX1* |
| Z | rs14768232 | 51601428 | A | 0.3125 | T | 2.328E-08 | *LNPEP* |
| Z | rs316782485 | 51606833 | A | 0.3125 | C | 2.328E-08 | *LNPEP* |
| Z | rs313126836 | 51607605 | A | 0.3125 | G | 2.328E-08 | *LNPEP* |
| Z | rs317785796 | 51612672 | A | 0.3125 | G | 2.328E-08 | *LNPEP* |
| Z | rs316397477 | 51614173 | A | 0.3125 | G | 2.328E-08 | *LNPEP* |
| Z | rs314836650 | 51619574 | C | 0.3125 | T | 2.328E-08 | *LNPEP* |
| Z | rs16114034 | 51625155 | A | 0.3125 | G | 2.328E-08 | *LNPEP* |
| Z | rs312887869 | 51634521 | T | 0.3125 | C | 2.328E-08 | *LNPEP* |
| Z | rs16770519 | 51637671 | T | 0.3125 | C | 2.328E-08 | *LNPEP* |
| Z | rs317679552 | 51639166 | C | 0.3125 | T | 2.328E-08 | *LNPEP* |
| Z | rs317495572 | 51640937 | G | 0.3125 | T | 2.328E-08 | *LNPEP* |
| Z | rs314929781 | 51643028 | T | 0.3125 | G | 2.328E-08 | *LNPEP* |
| Z | rs313222807 | 51648259 | T | 0.3125 | C | 2.328E-08 | *LNPEP* |
| Z | rs317635222 | 51652252 | T | 0.3125 | C | 2.328E-08 | *LNPEP* |
| Z | rs314945786 | 51657358 | C | 0.3125 | T | 2.328E-08 | *LNPEP* |
| Z | rs313745161 | 51660782 | A | 0.3125 | G | 2.328E-08 | *LNPEP* |
| Z | rs313930514 | 51665130 | T | 0.3125 | A | 2.328E-08 | *LNPEP* |
| Z | rs315077876 | 51669698 | T | 0.3125 | A | 2.328E-08 | *LNPEP* |
| Z | rs313642362 | 51682410 | G | 0.3125 | A | 2.328E-08 | *SHB* |
| Z | rs16770634 | 51690290 | G | 0.3125 | A | 2.328E-08 | *SHB* |
| Z | rs312427124 | 51697970 | C | 0.3125 | A | 2.328E-08 | *SHB* |
| Z | rs313213401 | 51705500 | C | 0.3125 | T | 2.328E-08 | *SHB* |
| Z | rs13768671 | 51714803 | T | 0.3125 | G | 2.328E-08 | *SHB* |
| Z | rs14768449 | 51721206 | T | 0.3125 | C | 2.328E-08 | *SHB* |
| Z | rs313527167 | 51748247 | A | 0.3125 | G | 2.328E-08 | *SHB* |
| Z | rs315850952 | 51748544 | G | 0.3125 | A | 2.328E-08 | *SHB* |
| Z | rs317385762 | 51748970 | A | 0.3125 | G | 2.328E-08 | *SHB* |
| Z | rs316942441 | 51754682 | T | 0.3125 | C | 2.328E-08 | *SHB* |
| Z | rs313944107 | 51758434 | T | 0.3125 | G | 2.328E-08 | *SHB* |
| Z | rs14768504 | 51759239 | G | 0.3125 | A | 2.328E-08 | *SHB* |
| Z | rs312805509 | 51766680 | A | 0.3125 | G | 2.328E-08 | *SHB* |
| Z | rs312876249 | 51770165 | C | 0.3125 | A | 2.328E-08 | *SHB* |
| Z | rs313216893 | 51772088 | T | 0.3125 | C | 2.328E-08 | *SHB* |
| Z | rs316662815 | 51775467 | A | 0.3125 | T | 2.328E-08 | *SHB* |
| Z | rs317217917 | 51779649 | A | 0.3125 | G | 2.328E-08 | *SHB* |
| Z | rs14768525 | 51788370 | T | 0.3125 | C | 2.328E-08 | *SHB* |
| Z | rs314439671 | 51791183 | A | 0.3125 | G | 2.328E-08 | *SHB* |
| Z | rs16770714 | 51792319 | C | 0.3125 | A | 2.328E-08 | *SHB* |
| Z | rs314239816 | 51797137 | T | 0.3125 | C | 2.328E-08 | *SHB* |
| Z | rs315795358 | 51803500 | A | 0.3125 | G | 2.328E-08 | *SHB* |
| Z | rs316810174 | 51812497 | C | 0.3125 | A | 2.328E-08 | *SHB* |
| Z | rs313689304 | 51815263 | C | 0.3125 | T | 2.328E-08 | *SHB* |
| Z | rs313726596 | 51819584 | A | 0.3125 | G | 2.328E-08 | *SHB* |
| Z | rs314177782 | 51830459 | T | 0.3125 | G | 2.328E-08 | *RNF38* |
| Z | rs312395587 | 51835322 | G | 0.3125 | A | 2.328E-08 | *RNF38* |
| Z | rs314709987 | 51843738 | A | 0.3125 | G | 2.328E-08 | *RNF38* |
| Z | rs16114273 | 51862104 | C | 0.3125 | A | 2.328E-08 | *RNF38* |
| Z | rs16770773 | 51865707 | A | 0.3125 | C | 2.328E-08 | *RNF38* |
| Z | rs16114279 | 51866394 | G | 0.3125 | A | 2.328E-08 | *RNF38* |
| Z | rs318218013 | 51875523 | C | 0.3125 | T | 2.328E-08 | *RNF38* |
| Z | rs317615410 | 51884339 | T | 0.3125 | C | 2.328E-08 | *RNF38* |
| Z | rs318052879 | 51898374 | C | 0.3125 | T | 2.328E-08 | *RNF38* |
| Z | rs315673618 | 51900000 | G | 0.3125 | A | 2.328E-08 | *RNF38* |
| Z | rs314358817 | 51904442 | C | 0.3125 | T | 2.328E-08 | *RNF38* |
| Z | rs313271499 | 51907658 | C | 0.3125 | A | 2.328E-08 | *RNF38* |
| Z | rs317814364 | 51910850 | G | 0.3125 | A | 2.328E-08 | *RNF38* |
| Z | rs317286363 | 51920910 | A | 0.3125 | T | 2.328E-08 | *RNF38* |
| Z | rs316731621 | 51923464 | C | 0.3125 | A | 2.328E-08 | *RNF38* |
| Z | rs316660594 | 51931473 | T | 0.3125 | C | 2.328E-08 | *RNF38* |
| Z | rs315726894 | 51932301 | C | 0.3125 | T | 2.328E-08 | *RNF38* |
| Z | rs316777780 | 51936167 | A | 0.3125 | C | 2.328E-08 | *RNF38* |
| Z | rs313070368 | 51943091 | G | 0.3125 | A | 2.328E-08 | *RNF38* |
| Z | rs317103307 | 51948363 | C | 0.3125 | T | 2.328E-08 | *RNF38* |
| Z | rs312276802 | 51959334 | C | 0.3125 | T | 2.328E-08 | *RNF38* |
| Z | rs313613337 | 51973797 | G | 0.3125 | A | 2.328E-08 | *RNF38* |
| Z | rs312794027 | 51976300 | T | 0.3125 | C | 2.328E-08 | *RNF38* |
| Z | rs14768635 | 51977339 | A | 0.3125 | G | 2.328E-08 | *RNF38* |
| Z | rs315139665 | 51978635 | A | 0.3125 | C | 2.328E-08 | *RNF38* |
| Z | rs313740484 | 51982202 | A | 0.3125 | G | 2.328E-08 | *RNF38* |
| Z | rs312433127 | 51988422 | C | 0.3125 | T | 2.328E-08 | *RNF38* |
| Z | rs14768666 | 51992658 | G | 0.3125 | A | 2.328E-08 | *RNF38* |
| Z | rs317366007 | 52005249 | G | 0.3125 | A | 2.328E-08 | *TRIM14* |
| Z | rs317624604 | 52005659 | T | 0.3125 | C | 2.328E-08 | *TRIM14* |
| Z | rs317226012 | 52011252 | A | 0.3125 | G | 2.328E-08 | *TRIM14* |
| Z | rs312804387 | 52017336 | G | 0.3125 | C | 2.328E-08 | *NANS* |
| Z | rs314275828 | 52020132 | C | 0.3125 | G | 2.328E-08 | *NANS* |
| Z | rs317776556 | 52026238 | G | 0.3125 | A | 2.328E-08 | *NANS* |
| Z | rs16706716 | 52030132 | G | 0.3125 | A | 2.328E-08 | *NANS* |
| Z | rs314557341 | 52036827 | G | 0.3125 | A | 2.328E-08 | *CLTA* |
| Z | rs318022861 | 52041796 | G | 0.3125 | A | 2.328E-08 | *CLTA* |
| Z | rs314984045 | 52059284 | T | 0.3125 | A | 2.328E-08 | *GNE* |
| Z | rs16114444 | 52061290 | C | 0.3125 | T | 2.328E-08 | *GNE* |
| Z | rs313156380 | 52062040 | A | 0.3125 | G | 2.328E-08 | *GNE* |
| Z | rs317719486 | 52064913 | G | 0.3125 | A | 2.328E-08 | *GNE* |
| Z | rs316623296 | 52068672 | G | 0.3125 | T | 2.328E-08 | *GNE* |
| Z | rs317758840 | 52071678 | T | 0.3125 | C | 2.328E-08 | *GNE* |
| Z | rs316717701 | 52075429 | G | 0.3125 | A | 2.328E-08 | *GNE* |
| Z | rs315416761 | 52090595 | C | 0.3125 | G | 2.328E-08 | *GNE* |
| Z | rs317782826 | 52091134 | G | 0.3125 | A | 2.328E-08 | *GNE* |
| Z | rs313768725 | 52100944 | G | 0.3125 | A | 2.328E-08 | *GNE* |
| Z | rs16114473 | 52104401 | G | 0.3125 | A | 2.328E-08 | *GNE* |
| Z | rs317926052 | 52105520 | T | 0.3125 | C | 2.328E-08 | *GNE* |
| Z | rs312692782 | 52111101 | G | 0.3125 | T | 2.328E-08 | *GNE* |
| Z | rs14768742 | 52113908 | G | 0.3125 | C | 2.328E-08 | *GNE* |
| Z | rs317348549 | 52119823 | C | 0.3125 | T | 2.328E-08 | *GNE* |
| Z | rs313726277 | 52123810 | A | 0.3125 | G | 2.328E-08 | *GNE* |
| Z | rs14768745 | 52125827 | T | 0.3125 | C | 2.328E-08 | *GNE* |
| Z | rs313797159 | 52130366 | G | 0.3125 | A | 2.328E-08 | *GNE* |
| Z | rs313525167 | 52132652 | A | 0.3125 | G | 2.328E-08 | *GNE* |
| Z | rs316649086 | 52134250 | G | 0.3125 | A | 2.328E-08 | *GNE* |
| Z | rs315912659 | 52143773 | A | 0.3125 | G | 2.328E-08 | *GNE* |
| Z | rs313583778 | 52144685 | T | 0.3125 | A | 2.328E-08 | *GNE* |
| Z | rs316786247 | 52146985 | T | 0.3125 | C | 2.328E-08 | *GNE* |
| Z | rs313024408 | 52150677 | C | 0.3125 | A | 2.328E-08 | *GNE* |
| Z | rs16114502 | 52155912 | T | 0.3125 | C | 2.328E-08 | *GNE* |
| Z | rs314347925 | 52160298 | G | 0.3125 | A | 2.328E-08 | *GNE* |
| Z | rs16770912 | 52160662 | G | 0.3125 | A | 2.328E-08 | *GNE* |
| Z | rs312818796 | 52163240 | C | 0.3125 | A | 2.328E-08 | *GNE* |
| Z | rs313410033 | 52171367 | C | 0.3125 | T | 2.328E-08 | *GNE* |
| Z | rs317229160 | 52173077 | C | 0.3125 | T | 2.328E-08 | *GNE* |
| Z | rs16770923 | 52175062 | C | 0.3125 | T | 2.328E-08 | *GNE* |
| Z | rs314203724 | 52176831 | C | 0.3125 | T | 2.328E-08 | *GNE* |
| Z | rs313756966 | 52182462 | G | 0.3125 | A | 2.328E-08 | *GNE* |
| Z | rs14768771 | 52184729 | C | 0.3125 | G | 2.328E-08 | *GNE* |
| Z | rs315925315 | 52189243 | A | 0.3125 | G | 2.328E-08 | *GNE* |
| Z | rs313132603 | 52194994 | A | 0.3125 | G | 2.328E-08 | *GNE* |
| Z | rs312990033 | 52197448 | T | 0.3125 | A | 2.328E-08 | *GNE* |
| Z | rs14768781 | 52200512 | C | 0.3125 | A | 2.328E-08 | *GNE* |
| Z | rs14768802 | 52206531 | G | 0.3125 | A | 2.328E-08 | *GNE* |
| Z | rs316496648 | 52211069 | A | 0.3125 | G | 2.328E-08 | *GNE* |
| Z | rs315148980 | 52212631 | G | 0.3125 | T | 2.328E-08 | *GNE* |
| Z | rs312566032 | 52218050 | C | 0.3125 | T | 2.328E-08 | *GNE* |
| Z | rs315732595 | 52222343 | T | 0.3125 | C | 2.328E-08 | *GNE* |
| Z | rs315738540 | 51801043 | G | 0.3191 | T | 2.465E-08 | *SHB* |
| Z | rs313634466 | 51439089 | T | 0.3158 | C | 4.241E-08 | *RIOK2* |
| Z | rs317224745 | 51946742 | A | 0.3158 | C | 4.241E-08 | *RNF38* |
| Z | rs316624046 | 51989605 | A | 0.3158 | G | 4.241E-08 | *RNF38* |
| Z | rs315211963 | 51459194 | C | 0.3053 | T | 4.683E-08 | *RIOK2* |
| Z | rs315993548 | 51657743 | T | 0.3053 | C | 4.683E-08 | *LNPEP* |
| Z | rs16770666 | 51735900 | T | 0.2979 | C | 4.683E-08 | *SHB* |
| Z | rs316791501 | 51884932 | G | 0.3053 | A | 4.683E-08 | *RNF38* |
| Z | rs316699281 | 51948004 | A | 0.3053 | G | 4.683E-08 | *RNF38* |
| Z | rs316211626 | 52103243 | C | 0.3053 | T | 4.683E-08 | *GNE* |
| Z | rs316869929 | 52117914 | T | 0.3053 | C | 4.683E-08 | *GNE* |
| Z | rs318014430 | 52164676 | G | 0.3053 | T | 4.683E-08 | *GNE* |
| Z | rs315275152 | 52052238 | G | 0.2979 | T | 9.497E-08 | *CLTA* |
| Z | rs316149223 | 50384840 | A | 0.2947 | G | 1.383E-07 | *SLCO4C1* |
| Z | rs14766656 | 50474037 | G | 0.2842 | A | 1.565E-07 | *ST8SIA4* |
| Z | rs316601199 | 50523221 | G | 0.2842 | A | 1.565E-07 | *ST8SIA4* |
| Z | rs315859788 | 52309321 | T | 0.3368 | C | 1.91E-07 | *CPLX1* |
| Z | rs317842530 | 52410193 | C | 0.3368 | T | 1.91E-07 | *CPLX1* |
| Z | rs316147137 | 50133832 | A | 0.2812 | G | 2.516E-07 | *PAM* |
| Z | rs313606898 | 50141153 | T | 0.2812 | C | 2.516E-07 | *PAM* |
| Z | rs313557983 | 50142560 | A | 0.2812 | G | 2.516E-07 | *PAM* |
| Z | rs318069119 | 50148396 | G | 0.2812 | A | 2.516E-07 | *PAM* |
| Z | rs316508272 | 50160689 | T | 0.2812 | C | 2.516E-07 | *PAM* |
| Z | rs312492749 | 50164393 | C | 0.2812 | G | 2.516E-07 | *PAM* |
| Z | rs313614131 | 50167774 | T | 0.2812 | C | 2.516E-07 | *PAM* |
| Z | rs317282198 | 50186022 | A | 0.2812 | C | 2.516E-07 | *PAM* |
| Z | rs16769640 | 50238117 | C | 0.2812 | G | 2.516E-07 | *SLCO4C1* |
| Z | rs316751547 | 50247066 | G | 0.2812 | C | 2.516E-07 | *SLCO4C1* |
| Z | rs315613443 | 50269624 | A | 0.2812 | G | 2.516E-07 | *SLCO4C1* |
| Z | rs316836609 | 50274008 | T | 0.2812 | C | 2.516E-07 | *SLCO4C1* |
| Z | rs313716861 | 50283914 | A | 0.2812 | G | 2.516E-07 | *SLCO4C1* |
| Z | rs317293515 | 50296958 | A | 0.2812 | G | 2.516E-07 | *SLCO4C1* |
| Z | rs313494937 | 50307617 | T | 0.2812 | C | 2.516E-07 | *SLCO4C1* |
| Z | rs314972036 | 50348490 | A | 0.2812 | T | 2.516E-07 | *SLCO4C1* |
| Z | rs314915984 | 50354438 | A | 0.2812 | G | 2.516E-07 | *SLCO4C1* |
| Z | rs313822679 | 50367373 | C | 0.2812 | T | 2.516E-07 | *SLCO4C1* |
| Z | rs13793817 | 50375628 | T | 0.2812 | C | 2.516E-07 | *SLCO4C1* |
| Z | rs313358167 | 50391495 | C | 0.2812 | T | 2.516E-07 | *SLCO4C1* |
| Z | rs16112762 | 50393698 | G | 0.2812 | T | 2.516E-07 | *SLCO4C1* |
| Z | rs314052445 | 50395032 | G | 0.2812 | A | 2.516E-07 | *SLCO4C1* |
| Z | rs317519663 | 50416281 | C | 0.2812 | A | 2.516E-07 | *SLCO4C1* |
| Z | rs317390044 | 50442485 | T | 0.2812 | C | 2.516E-07 | *SLCO4C1* |
| Z | rs314110578 | 50446109 | T | 0.2812 | A | 2.516E-07 | *SLCO4C1* |
| Z | rs317380983 | 50447706 | C | 0.2812 | T | 2.516E-07 | *SLCO4C1* |
| Z | rs315233522 | 50450968 | A | 0.2812 | G | 2.516E-07 | *SLCO4C1* |
| Z | rs315665886 | 50453261 | A | 0.2812 | G | 2.516E-07 | *SLCO4C1* |
| Z | rs312580343 | 50454448 | C | 0.2812 | A | 2.516E-07 | *SLCO4C1* |
| Z | rs14766668 | 50457538 | G | 0.2812 | A | 2.516E-07 | *SLCO4C1* |
| Z | rs316843012 | 50462565 | T | 0.2812 | G | 2.516E-07 | *SLCO4C1* |
| Z | rs316322840 | 50477502 | G | 0.2812 | A | 2.516E-07 | *ST8SIA4* |
| Z | rs16769432 | 50493805 | A | 0.2812 | T | 2.516E-07 | *ST8SIA4* |
| Z | rs16769419 | 50501830 | T | 0.2812 | C | 2.516E-07 | *ST8SIA4* |
| Z | rs314913097 | 50512603 | A | 0.2812 | G | 2.516E-07 | *ST8SIA4* |
| Z | rs317812756 | 50516075 | G | 0.2812 | T | 2.516E-07 | *ST8SIA4* |
| Z | rs316093048 | 50519646 | A | 0.2812 | G | 2.516E-07 | *ST8SIA4* |
| Z | rs313564210 | 50525083 | C | 0.2812 | T | 2.516E-07 | *ST8SIA4* |
| Z | rs14766608 | 50527122 | A | 0.2812 | G | 2.516E-07 | *ST8SIA4* |
| Z | rs314844028 | 50537008 | G | 0.2812 | A | 2.516E-07 | *ST8SIA4* |
| Z | rs316930230 | 50539984 | C | 0.2812 | T | 2.516E-07 | *ST8SIA4* |
| Z | rs313086999 | 50541253 | T | 0.2812 | C | 2.516E-07 | *ST8SIA4* |
| Z | rs313168423 | 50547695 | G | 0.2812 | A | 2.516E-07 | *ST8SIA4* |
| Z | rs316224643 | 50548087 | C | 0.2812 | A | 2.516E-07 | *ST8SIA4* |
| Z | rs317701171 | 50551325 | G | 0.2812 | A | 2.516E-07 | *ST8SIA4* |
| Z | rs312952114 | 50555952 | G | 0.2812 | A | 2.516E-07 | *ST8SIA4* |
| Z | rs314779449 | 50564117 | C | 0.2812 | T | 2.516E-07 | *ST8SIA4* |
| Z | rs314625761 | 50570328 | A | 0.2812 | G | 2.516E-07 | *ST8SIA4* |
| Z | rs16112549 | 50573827 | A | 0.2812 | G | 2.516E-07 | *ST8SIA4* |
| Z | rs13799767 | 50575747 | T | 0.2812 | A | 2.516E-07 | *ST8SIA4* |
| Z | rs314113459 | 50579442 | G | 0.2737 | A | 2.516E-07 | *ST8SIA4* |
| Z | rs312277758 | 50582895 | T | 0.2812 | C | 2.516E-07 | *ST8SIA4* |
| Z | rs316267705 | 50586202 | T | 0.2812 | C | 2.516E-07 | *ST8SIA4* |
| Z | rs316047202 | 50596695 | C | 0.2812 | T | 2.516E-07 | *ST8SIA4* |
| Z | rs317360368 | 50641383 | C | 0.2812 | T | 2.516E-07 | *FAM174A* |
| Z | rs313781232 | 50644086 | C | 0.2812 | T | 2.516E-07 | *FAM174A* |
| Z | rs14768881 | 52316895 | T | 0.3333 | C | 3.239E-07 | *CPLX1* |
| Z | rs14768884 | 52317267 | A | 0.3333 | G | 3.239E-07 | *CPLX1* |
| Z | rs312528685 | 52318796 | G | 0.3333 | A | 3.239E-07 | *CPLX1* |
| Z | rs312835634 | 52324728 | C | 0.3333 | G | 3.239E-07 | *CPLX1* |
| Z | rs16770997 | 52331765 | A | 0.3333 | G | 3.239E-07 | *CPLX1* |
| Z | rs313182818 | 52332430 | C | 0.3333 | T | 3.239E-07 | *CPLX1* |
| Z | rs317445360 | 52335470 | T | 0.3333 | C | 3.239E-07 | *CPLX1* |
| Z | rs313302660 | 52338594 | C | 0.3333 | T | 3.239E-07 | *CPLX1* |
| Z | rs312491232 | 52341427 | G | 0.3333 | A | 3.239E-07 | *CPLX1* |
| Z | rs317291164 | 52345948 | T | 0.3333 | A | 3.239E-07 | *CPLX1* |
| Z | rs314687259 | 52351243 | T | 0.3333 | C | 3.239E-07 | *CPLX1* |
| Z | rs317291295 | 52352231 | T | 0.3333 | G | 3.239E-07 | *CPLX1* |
| Z | rs14768910 | 52357498 | C | 0.3333 | A | 3.239E-07 | *CPLX1* |
| Z | rs14768912 | 52359682 | G | 0.3333 | A | 3.239E-07 | *CPLX1* |
| Z | rs318071284 | 52364194 | C | 0.3333 | T | 3.239E-07 | *CPLX1* |
| Z | rs313571069 | 52369600 | T | 0.3333 | C | 3.239E-07 | *CPLX1* |
| Z | rs314463958 | 52372980 | G | 0.3333 | A | 3.239E-07 | *CPLX1* |
| Z | rs16771024 | 52375075 | G | 0.3333 | T | 3.239E-07 | *CPLX1* |
| Z | rs315875389 | 52376061 | G | 0.3333 | T | 3.239E-07 | *CPLX1* |
| Z | rs317121174 | 52380681 | C | 0.3333 | G | 3.239E-07 | *CPLX1* |
| Z | rs318167844 | 52385259 | C | 0.3333 | A | 3.239E-07 | *CPLX1* |
| Z | rs316595820 | 52386169 | T | 0.3333 | A | 3.239E-07 | *CPLX1* |
| Z | rs16114700 | 52390788 | A | 0.3333 | G | 3.239E-07 | *CPLX1* |
| Z | rs314443272 | 52394250 | T | 0.3333 | C | 3.239E-07 | *CPLX1* |
| Z | rs312491571 | 52394503 | G | 0.3333 | T | 3.239E-07 | *CPLX1* |
| Z | rs312536114 | 52395228 | T | 0.3333 | C | 3.239E-07 | *CPLX1* |
| Z | rs313004205 | 52400269 | G | 0.3333 | A | 3.239E-07 | *CPLX1* |
| Z | rs317640423 | 52402524 | A | 0.3333 | G | 3.239E-07 | *CPLX1* |
| Z | rs316745834 | 52405913 | C | 0.3333 | T | 3.239E-07 | *CPLX1* |
| Z | rs314225506 | 52406708 | G | 0.3333 | T | 3.239E-07 | *CPLX1* |
| Z | rs313077015 | 52407436 | C | 0.3333 | T | 3.239E-07 | *CPLX1* |
| Z | rs315889212 | 52409581 | T | 0.3333 | C | 3.239E-07 | *CPLX1* |
| Z | rs312839182 | 52410626 | T | 0.3333 | C | 3.239E-07 | *CPLX1* |
| Z | rs317013453 | 52412024 | A | 0.3333 | G | 3.239E-07 | *CPLX1* |
| Z | rs313727449 | 52418080 | C | 0.3333 | T | 3.239E-07 | *CPLX1* |
| Z | rs14768966 | 52419121 | T | 0.3333 | A | 3.239E-07 | *CPLX1* |
| Z | rs316699827 | 52426105 | T | 0.3333 | C | 3.239E-07 | *CPLX1* |
| Z | rs315177475 | 52432923 | C | 0.3333 | G | 3.239E-07 | *CPLX1* |
| Z | rs313103697 | 52446176 | C | 0.3333 | T | 3.239E-07 | *CPLX1* |
| Z | rs313969106 | 52446694 | G | 0.3368 | T | 3.239E-07 | *CPLX1* |
| Z | rs316991498 | 52459856 | T | 0.3333 | C | 3.239E-07 | *CPLX1* |
| Z | rs313289924 | 52460616 | T | 0.3333 | C | 3.239E-07 | *CPLX1* |
| Z | rs318195279 | 52466864 | T | 0.3333 | A | 3.239E-07 | *CPLX1* |
| Z | rs313367373 | 52468355 | G | 0.3333 | A | 3.239E-07 | *CPLX1* |
| Z | rs314697188 | 52476503 | G | 0.3333 | A | 3.239E-07 | *CPLX1* |
| Z | rs314001929 | 52480176 | C | 0.3333 | T | 3.239E-07 | *CPLX1* |
| Z | rs317637017 | 52515304 | A | 0.3333 | G | 3.239E-07 | *CPLX1* |
| Z | rs315270308 | 50323420 | G | 0.2872 | A | 4.31E-07 | *SLCO4C1* |
| Z | rs313627542 | 50553512 | A | 0.2737 | G | 4.998E-07 | *ST8SIA4* |
| Z | rs317432717 | 52329108 | T | 0.3263 | A | 6.275E-07 | *CPLX1* |
| Z | rs14768895 | 52349802 | C | 0.3263 | T | 6.275E-07 | *CPLX1* |

^a^ Source: Reference Gallus_gallus-4.0 primary assembly (NCBI).
