# Supplementary material for: Genome-wide association study revealed genomic regions related to white/red earlobe color trait in the Rhode Island Red chickens
Source: BMC Genet. 2016 Aug 5;17:115. doi: 10.1186/s12863-016-0422-1 (PMC4974732; doi:10.1186/s12863-016-0422-1)
Supplement: Additional file 4: Table S2. — Suggestive SNPs associated with the earlobe color phenotype in Rhode Island Red chickens. (DOC 240 kb) [file 12863_2016_422_MOESM4_ESM.doc]

**Table S2. Suggestive SNPs associated with the earlobe color phenotype in Rhode Island Red chickens**

| SNP | Chromosome | Position^a^ (bp) | Minor allele | Major allele | MAF | *P* value |
| --- | --- | --- | --- | --- | --- | --- |
| rs317789654 | 31 | 52416054 | G | T | 0.3191 | 1.22E-06 |
| rs14766929 | 31 | 50212769 | T | C | 0.3895 | 1.94E-06 |
| rs312290334 | 31 | 50199984 | C | T | 0.3895 | 1.94E-06 |
| rs13799758 | 31 | 50847956 | C | A | 0.3895 | 1.94E-06 |
| rs316766012 | 31 | 52454293 | G | A | 0.3021 | 3.08E-06 |
| rs312775311 | 31 | 52493378 | T | C | 0.3021 | 3.08E-06 |
| rs312758963 | 31 | 52452348 | C | T | 0.3021 | 3.08E-06 |
| rs314571155 | 31 | 52499019 | T | C | 0.3021 | 3.08E-06 |
| rs312659355 | 31 | 52443587 | C | G | 0.3021 | 3.08E-06 |
| rs312556115 | 31 | 52499741 | T | C | 0.3021 | 3.08E-06 |
| rs317608513 | 31 | 52502401 | G | A | 0.3021 | 3.08E-06 |
| rs314364303 | 31 | 52510108 | C | T | 0.3021 | 3.08E-06 |
| rs317966573 | 31 | 52510957 | C | T | 0.3021 | 3.08E-06 |
| rs16114744 | 31 | 52441375 | G | C | 0.3021 | 3.08E-06 |
| rs14769053 | 31 | 52521636 | G | C | 0.3125 | 3.08E-06 |
| rs14716129 | 31 | 48145988 | G | T | 0.2917 | 3.08E-06 |
| rs316622670 | 31 | 52572624 | G | A | 0.3125 | 3.08E-06 |
| rs316745358 | 31 | 48142801 | T | C | 0.2917 | 3.08E-06 |
| rs314740416 | 31 | 48140819 | A | G | 0.2917 | 3.08E-06 |
| rs314870403 | 31 | 48317675 | A | G | 0.2917 | 3.08E-06 |
| rs313871159 | 31 | 48233969 | T | G | 0.2917 | 3.08E-06 |
| rs313819897 | 31 | 52593201 | T | C | 0.3125 | 3.08E-06 |
| rs313999500 | 31 | 48132739 | C | T | 0.2917 | 3.08E-06 |
| rs312531603 | 31 | 48127175 | T | A | 0.2917 | 3.08E-06 |
| rs16771133 | 31 | 52602670 | A | G | 0.3125 | 3.08E-06 |
| rs14769160 | 31 | 52612760 | C | T | 0.3125 | 3.08E-06 |
| rs16771155 | 31 | 52623483 | C | T | 0.3125 | 3.08E-06 |
| rs312470556 | 31 | 52624240 | G | A | 0.3125 | 3.08E-06 |
| rs16771164 | 31 | 52625813 | G | C | 0.3125 | 3.08E-06 |
| rs316719801 | 31 | 52635659 | A | G | 0.3125 | 3.08E-06 |
| rs315574236 | 31 | 52637151 | A | G | 0.3125 | 3.08E-06 |
| rs315480814 | 31 | 52638788 | C | A | 0.3125 | 3.08E-06 |
| rs317200314 | 31 | 52644387 | A | G | 0.3125 | 3.08E-06 |
| rs16771168 | 31 | 52644784 | G | A | 0.3125 | 3.08E-06 |
| rs314783595 | 31 | 52647240 | C | A | 0.3125 | 3.08E-06 |
| rs14769200 | 31 | 52654051 | C | T | 0.3125 | 3.08E-06 |
| rs314725846 | 31 | 52654646 | G | A | 0.3125 | 3.08E-06 |
| rs315386334 | 31 | 52659901 | C | T | 0.3125 | 3.08E-06 |
| rs312433497 | 31 | 52665631 | T | G | 0.3125 | 3.08E-06 |
| rs314869585 | 31 | 52669284 | A | G | 0.3125 | 3.08E-06 |
| rs14769218 | 31 | 52670254 | A | G | 0.3125 | 3.08E-06 |
| rs313320355 | 31 | 52672605 | G | A | 0.3125 | 3.08E-06 |
| rs313148876 | 31 | 52718617 | A | T | 0.3125 | 3.08E-06 |
| rs314992334 | 31 | 52722576 | A | G | 0.3125 | 3.08E-06 |
| rs315983580 | 31 | 52726534 | A | T | 0.3125 | 3.08E-06 |
| rs316081572 | 31 | 52773783 | C | T | 0.3125 | 3.08E-06 |
| rs317748659 | 31 | 52785024 | C | T | 0.3125 | 3.08E-06 |
| rs14769309 | 31 | 52788783 | T | C | 0.3125 | 3.08E-06 |
| rs16689428 | 31 | 48330433 | C | T | 0.2917 | 3.08E-06 |
| rs14769311 | 31 | 52789892 | G | A | 0.3125 | 3.08E-06 |
| rs318148424 | 31 | 52828254 | G | A | 0.3125 | 3.08E-06 |
| rs14769342 | 31 | 52830701 | T | C | 0.3125 | 3.08E-06 |
| rs317177679 | 31 | 48263419 | T | C | 0.2917 | 3.08E-06 |
| rs316440465 | 31 | 52847692 | G | A | 0.3125 | 3.08E-06 |
| rs317942314 | 31 | 48310197 | A | G | 0.2947 | 3.08E-06 |
| rs313608161 | 31 | 52856739 | T | G | 0.3125 | 3.08E-06 |
| rs313664083 | 31 | 52861596 | C | T | 0.3125 | 3.08E-06 |
| rs316433815 | 31 | 48340320 | C | T | 0.2917 | 3.08E-06 |
| rs316313536 | 31 | 52876363 | T | C | 0.3125 | 3.08E-06 |
| rs316059270 | 31 | 52885158 | T | A | 0.3125 | 3.08E-06 |
| rs313036468 | 31 | 52900431 | G | A | 0.3125 | 3.08E-06 |
| rs313012836 | 31 | 52913544 | G | T | 0.3125 | 3.08E-06 |
| rs14769029 | 31 | 52496443 | T | G | 0.3021 | 3.08E-06 |
| rs313161090 | 31 | 52834097 | G | C | 0.3125 | 3.08E-06 |
| rs315709370 | 31 | 48345469 | T | C | 0.2917 | 3.08E-06 |
| rs317332884 | 31 | 52929780 | A | G | 0.3125 | 3.08E-06 |
| rs14769465 | 31 | 52996635 | G | A | 0.3125 | 3.08E-06 |
| rs314923150 | 31 | 48356232 | C | T | 0.2917 | 3.08E-06 |
| rs316452039 | 31 | 48397717 | T | G | 0.2917 | 3.08E-06 |
| rs317095228 | 31 | 48456749 | C | T | 0.2842 | 3.08E-06 |
| rs14769569 | 31 | 53138429 | C | T | 0.3125 | 3.08E-06 |
| rs14769694 | 31 | 53284238 | C | T | 0.3125 | 3.08E-06 |
| rs312898992 | 31 | 48552057 | G | A | 0.2917 | 3.08E-06 |
| rs315885894 | 31 | 50127870 | G | A | 0.2917 | 3.08E-06 |
| rs313136067 | 31 | 50099464 | T | A | 0.2917 | 3.08E-06 |
| rs313313306 | 31 | 50090655 | C | T | 0.2917 | 3.08E-06 |
| rs313028881 | 31 | 50085523 | C | T | 0.2917 | 3.08E-06 |
| rs314362138 | 31 | 50075909 | A | C | 0.2917 | 3.08E-06 |
| rs312755064 | 31 | 50072308 | T | C | 0.2917 | 3.08E-06 |
| rs16112934 | 31 | 50061827 | C | A | 0.2917 | 3.08E-06 |
| rs317909895 | 31 | 50045957 | C | T | 0.2917 | 3.08E-06 |
| rs312314941 | 31 | 50032513 | G | T | 0.2917 | 3.08E-06 |
| rs315573982 | 31 | 50030505 | G | A | 0.2917 | 3.08E-06 |
| rs315202096 | 31 | 50029347 | G | A | 0.2917 | 3.08E-06 |
| rs315900904 | 31 | 50018632 | C | G | 0.2917 | 3.08E-06 |
| rs317336958 | 31 | 50013938 | A | G | 0.2917 | 3.08E-06 |
| rs14767136 | 31 | 50007141 | C | T | 0.2917 | 3.08E-06 |
| rs317513136 | 31 | 50000564 | T | A | 0.2917 | 3.08E-06 |
| rs317595119 | 31 | 49865998 | G | A | 0.2917 | 3.08E-06 |
| rs314006557 | 31 | 49854339 | A | G | 0.2917 | 3.08E-06 |
| rs317541608 | 31 | 49853117 | C | T | 0.2917 | 3.08E-06 |
| rs14767304 | 31 | 49847675 | T | C | 0.2917 | 3.08E-06 |
| rs315604081 | 31 | 49846262 | G | A | 0.2917 | 3.08E-06 |
| rs14767332 | 31 | 49828937 | G | A | 0.2917 | 3.08E-06 |
| rs314800627 | 31 | 49827958 | G | A | 0.2917 | 3.08E-06 |
| rs316430062 | 31 | 49821486 | A | C | 0.2917 | 3.08E-06 |
| rs315024799 | 31 | 49817569 | G | A | 0.2917 | 3.08E-06 |
| rs315809606 | 31 | 49799955 | A | G | 0.2917 | 3.08E-06 |
| rs317474149 | 31 | 49785489 | G | A | 0.2917 | 3.08E-06 |
| rs313530016 | 31 | 49781647 | A | G | 0.2917 | 3.08E-06 |
| rs14767394 | 31 | 49778443 | C | T | 0.2917 | 3.08E-06 |
| rs313242147 | 31 | 49776993 | C | T | 0.2917 | 3.08E-06 |
| rs314854125 | 31 | 49768509 | A | G | 0.2917 | 3.08E-06 |
| rs317463096 | 31 | 49673836 | T | C | 0.2917 | 3.08E-06 |
| rs314021841 | 31 | 49670299 | A | C | 0.2917 | 3.08E-06 |
| rs316947890 | 31 | 49659168 | T | A | 0.2917 | 3.08E-06 |
| rs317732139 | 31 | 49656296 | T | A | 0.2917 | 3.08E-06 |
| rs313040454 | 31 | 49653421 | A | G | 0.2917 | 3.08E-06 |
| rs314090185 | 31 | 49644298 | T | C | 0.2917 | 3.08E-06 |
| rs313042766 | 31 | 49639347 | G | A | 0.2917 | 3.08E-06 |
| rs14767459 | 31 | 49635546 | C | T | 0.2917 | 3.08E-06 |
| rs14767469 | 31 | 49631031 | A | G | 0.2917 | 3.08E-06 |
| rs317839506 | 31 | 49625136 | G | T | 0.2917 | 3.08E-06 |
| rs317406654 | 31 | 49623105 | A | G | 0.2917 | 3.08E-06 |
| rs315664700 | 31 | 49618553 | G | T | 0.2917 | 3.08E-06 |
| rs315088529 | 31 | 49618379 | A | G | 0.2917 | 3.08E-06 |
| rs315144219 | 31 | 49554828 | G | T | 0.2917 | 3.08E-06 |
| rs312363936 | 31 | 49535005 | A | G | 0.2917 | 3.08E-06 |
| rs314948182 | 31 | 49518012 | G | T | 0.2917 | 3.08E-06 |
| rs314750597 | 31 | 49476608 | G | A | 0.2917 | 3.08E-06 |
| rs14767540 | 31 | 49475926 | A | G | 0.2917 | 3.08E-06 |
| rs316919860 | 31 | 49475235 | A | T | 0.2917 | 3.08E-06 |
| rs16769971 | 31 | 49474371 | A | G | 0.2917 | 3.08E-06 |
| rs312400243 | 31 | 49312336 | A | G | 0.2917 | 3.08E-06 |
| rs317078074 | 31 | 49428707 | T | C | 0.2917 | 3.08E-06 |
| rs314142315 | 31 | 49428135 | C | A | 0.2917 | 3.08E-06 |
| rs315961154 | 31 | 49426340 | C | T | 0.2917 | 3.08E-06 |
| rs14769847 | 31 | 53521506 | C | T | 0.3438 | 3.41E-06 |
| rs312277011 | 31 | 53980250 | T | A | 0.3438 | 3.41E-06 |
| rs14769356 | 31 | 52872959 | G | A | 0.3158 | 5.19E-06 |
| rs314903534 | 31 | 52920684 | C | T | 0.3158 | 5.19E-06 |
| rs314883226 | 31 | 52597262 | T | C | 0.3158 | 5.19E-06 |
| rs14769141 | 31 | 52588096 | C | T | 0.3073 | 5.25E-06 |
| rs317255483 | 31 | 48314960 | T | C | 0.2842 | 5.86E-06 |
| rs14769118 | 31 | 52576991 | C | G | 0.3053 | 5.86E-06 |
| rs14536112 | 5 | 38631036 | T | C | 0.3947 | 7.14E-06 |
| rs315778412 | 31 | 47967217 | A | G | 0.2812 | 8.86E-06 |
| rs318175446 | 31 | 47974219 | A | G | 0.2812 | 8.86E-06 |
| rs315506420 | 31 | 47975064 | T | A | 0.2812 | 8.86E-06 |
| rs317661054 | 31 | 48036718 | C | G | 0.2812 | 8.86E-06 |
| rs315239803 | 31 | 48038035 | A | T | 0.2812 | 8.86E-06 |
| rs312706680 | 31 | 48067046 | T | C | 0.2812 | 8.86E-06 |
| rs312785501 | 31 | 48047795 | A | G | 0.2812 | 8.86E-06 |
| rs313771706 | 31 | 48054281 | A | G | 0.2812 | 8.86E-06 |
| rs317569167 | 31 | 48040581 | A | G | 0.2812 | 8.86E-06 |
| rs312386721 | 2 | 125354703 | G | A | 0.4511 | 1.13E-05 |
| rs315420052 | 2 | 67196938 | A | G | 0.2448 | 1.32E-05 |
| rs313803643 | 2 | 67209022 | C | A | 0.2448 | 1.32E-05 |
| rs316577182 | 7 | 10095418 | A | G | 0.4167 | 1.56E-05 |
| rs14769107 | 31 | 52571034 | C | T | 0.2842 | 1.68E-05 |
| rs314012571 | 31 | 52789747 | A | G | 0.2842 | 1.68E-05 |
| rs317653918 | 31 | 53049154 | T | A | 0.2842 | 1.68E-05 |
| rs312900954 | 31 | 52584785 | C | T | 0.2842 | 1.68E-05 |
| rs317494737 | 7 | 10111401 | A | G | 0.375 | 1.70E-05 |
| rs313424821 | 31 | 53867977 | C | T | 0.3158 | 1.84E-05 |

^a^ Position for SNPs according to the reference Gallus_gallus-4.0 primary assembly.
